# Trauma-induced large true superficial femoral artery aneurysm: A case report

**DOI:** 10.1016/j.amsu.2020.05.020

**Published:** 2020-05-30

**Authors:** Firas shaker Mahmoud Al-Faham, Samer Makki Mohamed Al-Hakkak, Mehmet Besir Akpinar

**Affiliations:** a Department of Surgery, College of Medicine, Kerbala University, Kerbala City, Iraq; b Department of Surgery, Faculty of Medicine, Jabir ibn Hayyan Medical University, Najaf City, Iraq; c Al Kafeel Hospital, Kerbala City, Iraq

**Keywords:** True superficial femoral artery aneurysm, Trauma, Prosthetic vascular repair, Arterio-venous fistula, Vascular injury

## Abstract

**Background:**

A femoral aneurysm is a weakness and bulging in the femoral artery wall located in the thigh. Femoral aneurysms can burst, which may cause uncontrolled bleeding and life-threatening conditions. The aneurysm may also cause a blood clot, showering emboli, potentially resulting in leg ischemia and amputation.

**Case report:**

A 49-year-old man with hypertension presented significant swelling in his right thigh. The patient had a history of surgery for arteriovenous fistula repair. The arteriovenous fistula in the thigh was caused by a bullet injury during the war. Diagnosis of the superficial femoral artery aneurysm was determined using magnetic resonance angiogram. The aneurysm was surgically excised and a prosthetic vascular graft was inserted.

**Discussion:**

The exact cause of femoral aneurysms is unknown, although atherosclerosis and hypertension may play a key role. Trauma to the artery may also be a contributing factor. Long-standing occult arteriovenous fistula plays a significant role in the cause of distal aneurysms.

**Conclusion:**

Femoral aneurysms are usually treated surgically. A surgeon will replace the artery with a graft or create a bypass around the area of the artery where the aneurysm is present.

## Highlights

• Traumatic bullet thigh injury need follow up even asymptomatic.
• Hypertension may lead to an aneurysm, especially in the vulnerable artery.
• Traumatic induced AVF lead to serious complication if discovered late.
• CT angio non-beneficial in large aneurysm because of rapid disappear of dye.
• MRA right choice for diagnosis and evaluation of giant SFA aneurysm.

## 1 Introduction

Peripheral arterial aneurysms are rare, and superficial femoral arterial (SFA) aneurysms are sporadic [[1], [2], [3]]. SFA aneurysms are not associated with any typical symptoms, and their early diagnosis is difficult. These aneurysms have a high rate of rupture, and surgery plays a vital role in their treatment. However, standard methods have not yet been established because of the rarity of SFA aneurysms [[3], [4], [5], [6]]. They occur in older men, often with other manifestations of atherosclerosis. One third is bilateral, and nearly two-thirds are associated with aneurysms elsewhere (e.g., popliteal and aortic). SFAs can reach a large size, and symptoms such as limb-threatening ischemia, embolization, or tissue loss may occur; however, rupture rarely occurs. SFA is often asymptomatic. Local pain, distal embolization, crack, and venous compression may all be presenting features. We describe a case of an SFA aneurysm in a patient who was successfully treated with an artificial blood vessel graft. The work has been reported in line with the SCARE [7].

## 2 Presentation of case

**Fig. 1:**
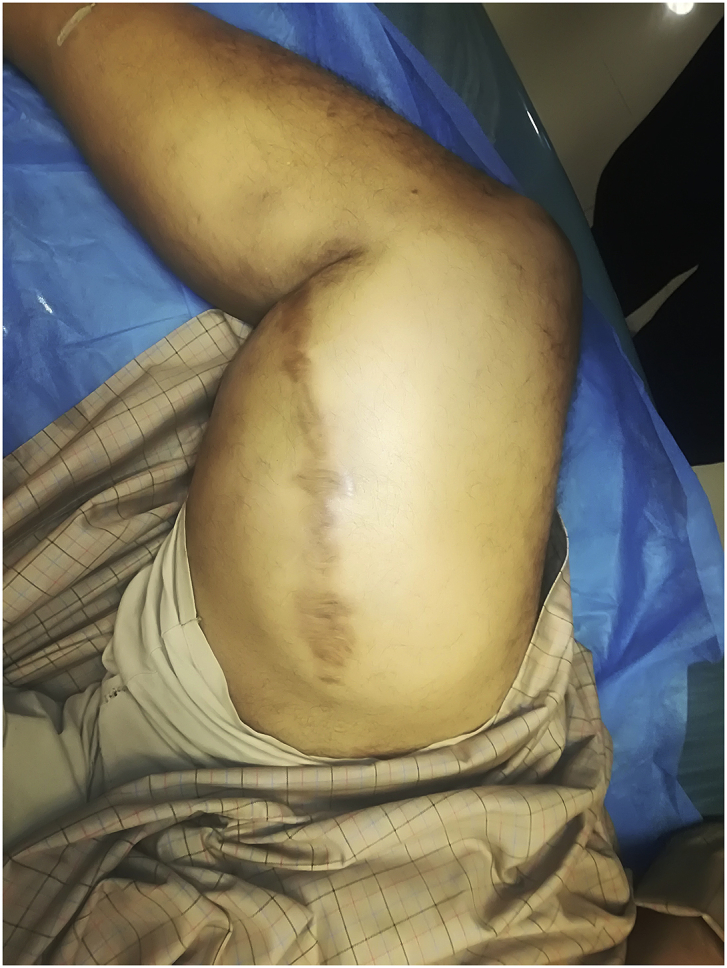
Early presentation of right thigh swelling.

During the Gulf War in 1991, a 20-year-old soldier in the Iraqi army was subjected to a bullet injury in the right thigh near the knee joint. Bleeding at the site of the injury occurred without signs and symptoms of ischemia. The patient was transferred to a military hospital after dressing and applying firm bandaging. However, the bleeding stopped upon arrival. Because of many causes, the patient was treated conservatively for five days by changing the dressing, applying an antibiotic cover, observation, and discharge without vascular assessment and duplex ultrasound. After many years, the patient experienced shortness of breath and was easily fatigued, but the patient did not seek medical attention because he lived in a rural area and had financial problems. In 2004, the patient consulted a local general practitioner doctor because he developed swelling in his right thigh, had dilated superficial veins and abdominal wall, and experienced dyspnea and fatigability. The symptoms were severe enough that walking and work became difficult. which examined him and found thrill all over the right thigh and anterior abdominal wall with positive dorsalis pedis and posterior tibial pulse. In addition, bilateral basal crepitation and bilateral leg edema were diagnosed. The patient underwent a duplex ultrasound, which revealed a single arteriovenous fistula (AVF) between the proximal popliteal artery and vein. The patient was sent to a specialized tertiary hospital, after full investigation and echo study, which showed the features of heart failure. Subsequently, the patient underwent computerized tomography angiography (CTA), which confirmed the diagnosis of an AV fistula. The patient prepared for surgery, and exploration of the femopopletal artery and vein was performed after establishing proximal control of the common femoral artery. The artery and vein were isolated, and repair of the popliteal vein began with the use of a Dacron interposition graft (10 cm length) to replace the diseased popliteal artery. The patient had a smooth postoperative period and started walking two days after surgery with positive distal pulses. The thrills disappeared from the area immediately after the operation. He was then administered oral antiplatelets for three months postoperatively. During the first six months postoperatively, all symptoms of heart failure disappeared, and the patient recovered. In 2019, the patient suddenly developed pain, pulsatile swelling of the right thigh, and a cold foot. The patient returned to the same surgeon to seek medical advice. On examination, there was pulsatile swelling involving most of the right thigh, but no thrill over the area, with negative distal pulses, as shown in Fig. 1. In addition, the patient had a history of uncontrolled hypertension within the last 10 years. The patient underwent CTA, and it was non-conclusive because the contrast rapidly disappeared in the large sack. Magnetic resonance angiography showed a massive 10× 10 × 15-cm true aneurysm of the SFA and the old graft compressed by a large aneurysm and inadequate blood supply to the distal part of the leg, as shown in Fig. 2a, Fig. 2ba and b. His medical history of hypertension included treatment with Diovan 160 mg once daily, but blood pressure control before surgery, complete investigation, and preparation of four units of blood and five units of plasma were required before urgent surgery proceeded. Under general anasthesia and in the supine position, sterilization and draping the area were initiated to begin the operation by proximal control of the common femoral artery, which dilates. The aneurysm was explored using a longitudinal incision that showed massive thrombosis inside the sac and bleeding from the inside branches that were sutured and stop bleeding. The old graft that was compressed was also excised and removed entirely. Fresh end of the popliteal artery was prepared. Anastomosis then a 40 cm Dacron graft size 8 mm proximally to the beginning of SFA and distally to the popliteal artery, as shown in Fig. (3) The incision was closed by using layers properly over a ready-vac drain. Postoperatively, the patient was administered enoxaparin and analgesia. Five days after surgery, the patient was discharged but continued oral antiplatelet therapy (aspirin 100 mg and Plavix 75 mg). One month later, CTA showed good flow in the common femoral Dacron graft and popliteal artery as shown in Fig. 4a, Fig. 4ba and b. The stitches were removed two weeks later as shown in Fig. 5. The patient was monitored strictly during follow-up by receiving monthly Doppler ultrasound and other investigations and instructions about continuing his medication. To date, he has been observed for six months of follow-up. We recommended monthly follow-up for one year. If the patient's condition is good, then we can increase the duration of follow-up by 3–4 months in the second year for early detection of any complication.

**Fig. 2a:**
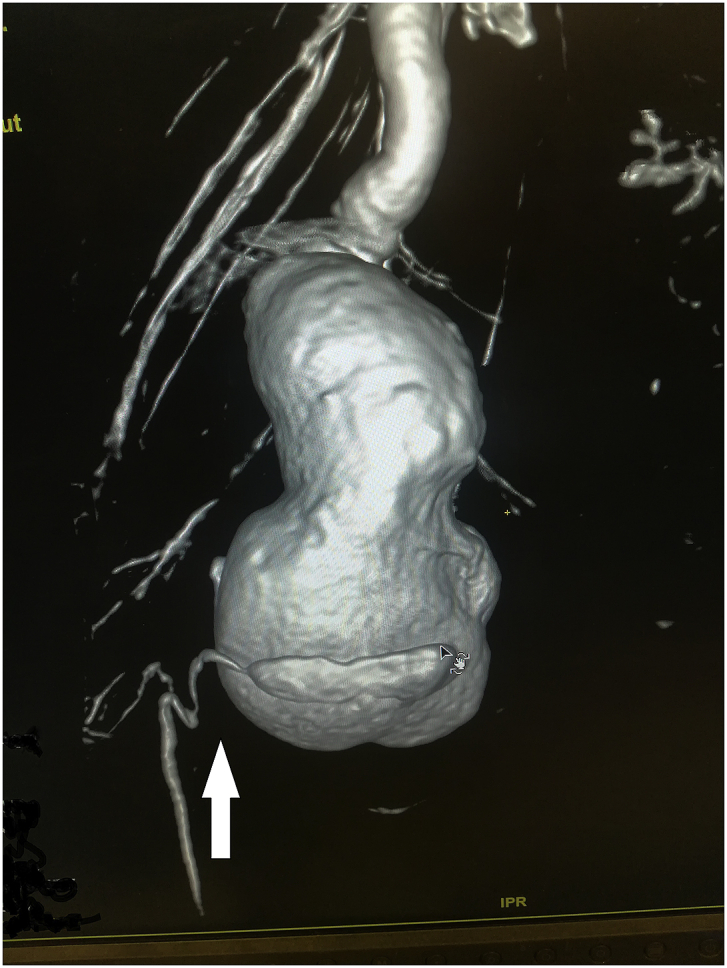
MRA show SFA aneurysm, constriction by previous graft.

**Fig. 2b:**
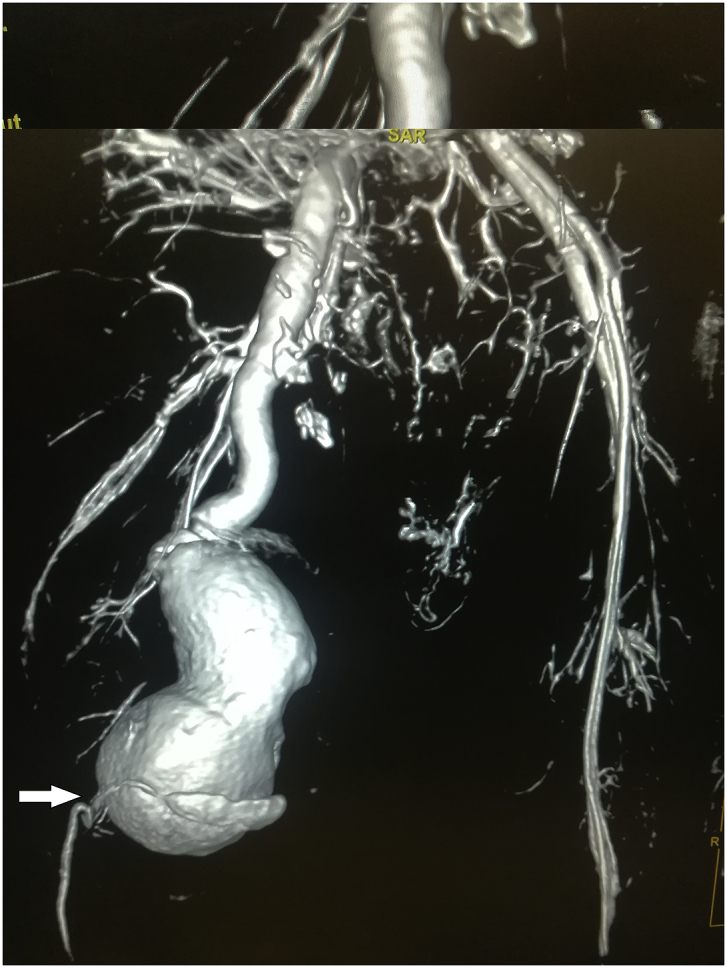
MRA show true SFA, distal dilated artery due to long standing compression.

**Fig. 3:**
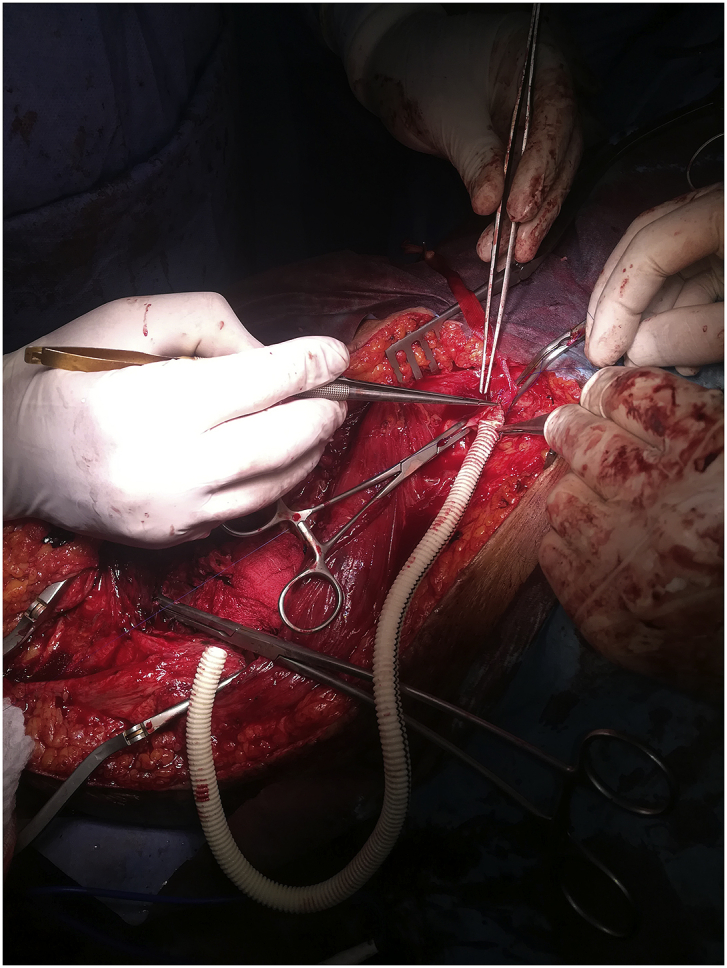
Suturing of synthetic graft.

**Fig. 4a:**
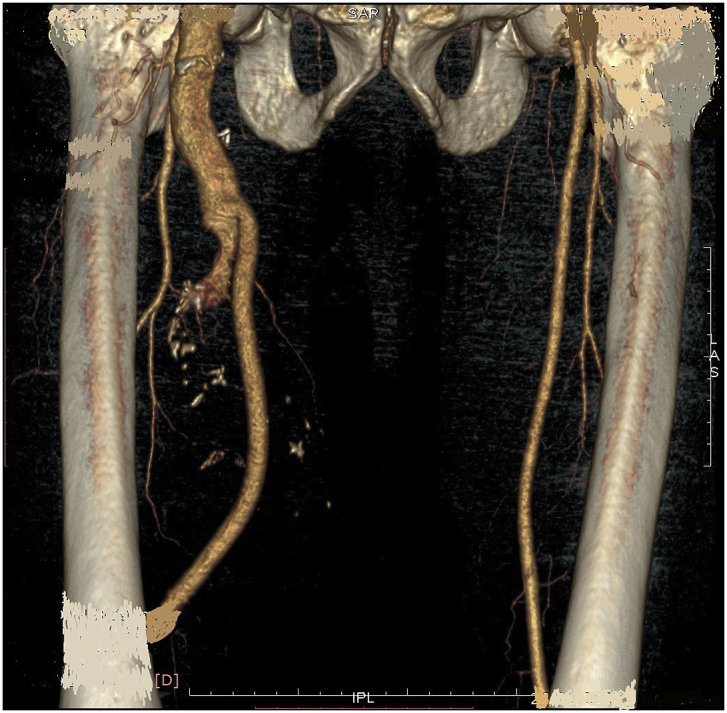
CT angio post operative.

**Fig. 4b:**
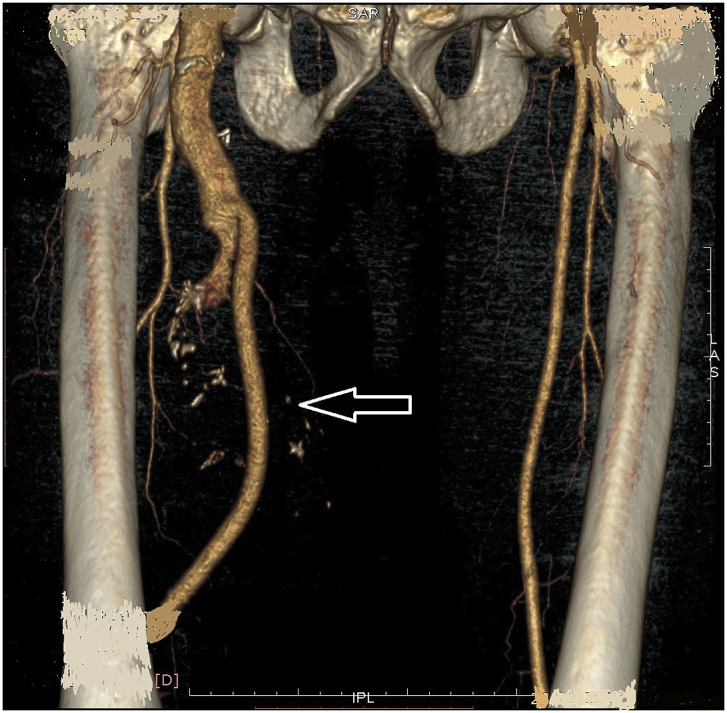
CTA post-operative,arrow show new anastomosis graft.

**Fig. 5:**
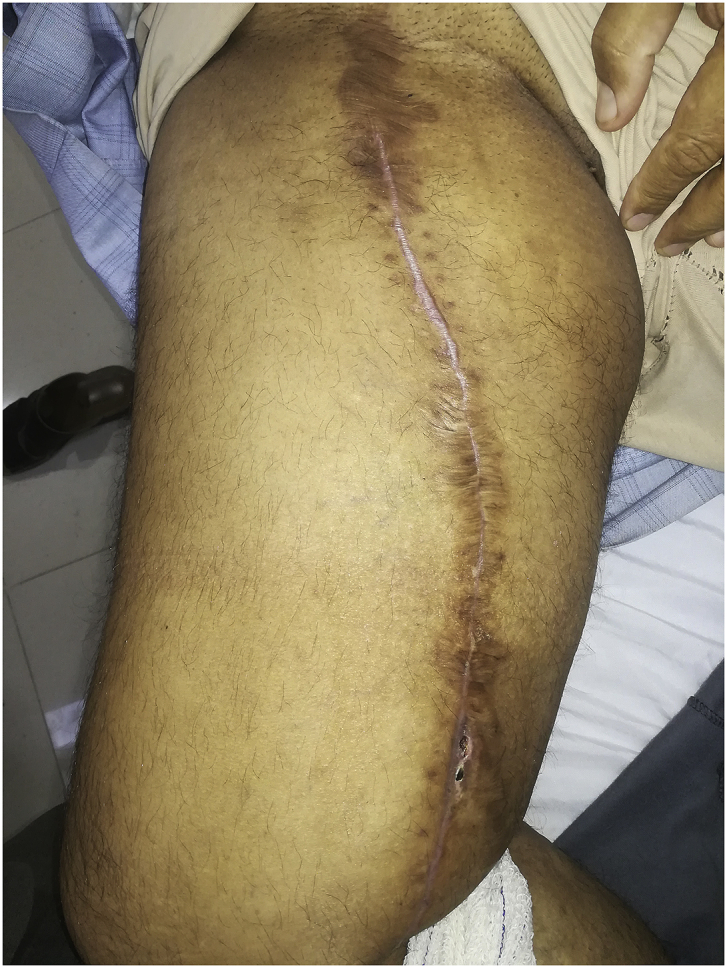
Post operative surgery stitch removal.

## 3 Discussion

Femoral aneurysms are uncommon and frequently associated with other aneurysms, particularly those of the aorta and popliteal arteries. True isolated atherosclerotic aneurysms of the superficial femoral artery are a rare pathology [7]. True femoral artery aneurysms are attributed to weakness in the arterial wall, which is usually formed by atherosclerosis. Our patient was subjected to a bullet injury during war that had entrance and exit in the lower right thigh. This lead to the development of AVF, trauma, especially low-velocity wound from a knife or small-caliber missile, is a major cause of acquired AVF. Approximately 2% of post-traumatic AVFs spontaneously resolve [8]. Increased sheer force from shunt flow in the artery proximal to the fistula stimulates secretion of the endothelium-derived relaxation factor, resulting in vasodilatation by its action on the arterial wall smooth muscle. Long-standing increased blood flow will increase the diameter of the vessel and enhance fracture of the elastic fibers, further dilating the vessel and increasing shunt flow [9]. With the enlarging left-to-right shunt, chronic volume overload of the heart leads to remodeling, ventricular dilatation, and heart failure. Such as in this patient, it is difficult to diagnose at the time of injury because of the lack of symptoms; further, this patient was sent home after stabilization of his condition without bleeding and intact distal pulses. Fourteen years after a war injury, the patient presented with swelling of the right thigh, thrill over the entire thigh and lower abdomen with signs and symptoms of heart failure. Duplex revealed a large AVF between the popliteal artery and vein. Surgery was performed to repair the vein and the use of a synthetic arterial graft replaced the diseased arteries. This prolonged standing fistula caused weakness of the superficial femoral, common femoral, and external iliac arteries. Post-traumatic fistulas should obliterate surgically as soon as possible [10]. Untreated fistulas result in complications, including rennin-mediated hypertension (40%−50%) and high-output heart failure (50%) [11]. The patient was lost during follow-up and returned after 15 years with swelling of the right thigh and symptoms of a large SFA aneurysm caused by an already weakened SFA wall and because of the presence of a distal popliteal graft. The high local blood pressure at the site of the distal part of the superficial femoral artery caused such a large aneurysm after being in place for 15 years. The force of blood as it passes through blood vessels is measured by how much pressure it places on your artery walls. If the pressure increases above a standard rate, it may enlarge or weaken the blood vessels. Aneurysmal dilatation can occur naturally over time because of the increased blood flow, and the process may accelerate by raising the pressure within the fistula. A large aneurysm of the SFA might lead to life-threatening pulmonary thromboembolism, and some authors suggest aggressive surgical correction [12]. Delays in the diagnosis and treatment of traumatic AVF may lead to many complications, including heart failure, hypertension, diseased artery and aneurysm, venous hypertension, and venous insufficiency. In the event of an injury or suspicion of vascular injury, it should be thoroughly monitored and checked because of the subsequent problems that may entail, as what occurred in our case.

## 4 Conclusion

Superficial femoral artery aneurysms generally require surgical repair if left untreated. Aneurysms lead to a risk of rupture and severe bleeding, which can be fatal due to exsanguination. A traumatic arteriovenous fistula is one of the reversible causes of congestive heart failure. Although the history of trauma is clear, the development of AVF is insidious and sometimes not obvious; thus, the detection of the shunt is crucial for treatment. Any patient with a bullet injury to the upper or lower extremities, even without bleeding or hematoma, should be subjected to accurate vascular assessment and duplex ultrasound or even CTA to prevent missing cases of vascular injuries.

## Ethical approval

Ethical committee of Faculty of Medicine/Jabir Ibn Hayyan Medical University.

## Funding

Nil.

## Author contribution

Firas Shaker Mahmoud Al-Faham.

Vascular surgeon who perform the interventions, collection of data, the conception and design of the study, or acquisition of data, or analysis and interpretation of data.

Samer Makki Mohamed Al-Hakkak.

Data analysis and interpretation and writing the paper.

Drafting the article or revising it critically for important intellectual content.

Final approval of the version to be submitted.

Mehmet Besir Akpinar.

Vascular surgeon who assist in second surgery.

## Trial registry number

1. Researchregistery

2. Researchregistry5471.

3. https://www.researchregistry.com/browse-the-registry.

## Guarantor

Dr Samer Makki Mohamed Al-Hakkak.

## Consent

Taken from the patient and his family.

## Provenance and peer review

Not commissioned, externally peer reviewed.

## Declaration of competing interest

Nil.

## References

[1] Roseman J.M., Wyche D. (1987). True aneurysm of the profunda femoris artery. Literature review, differential diagnosis, management. J. Cardiovasc. Surg..

[2] Johnson C.A., Goff J.M., Rehrig S.T., Hadro N.C. (2002). Asymptomatic profunda femoris artery aneurysm: diagnosis and rationale for management. Eur. J. Vasc. Endovasc. Surg..

[3] Toda R., Yuda T., Watanabe S., Hisashi Y., Moriyama Y., Taira A. (2000). Surgical repair of a solitary deep femoral arterial aneurysm: report of two cases. Surg. Today.

[4] Maruyama Y., Ochi M., Shimizu K. (2012). Surgical management of a deep femoral artery aneurysm. J. Nippon Med. Sch..

[5] Hariharan D., Singhal R., Bahal V. (2006). Deep femoral artery aneurysm: report of a case. Surg. Today.

[6] Cho Y.P., Choi S.J., Kwon T.W., Han M.S., Kim Y.H., Kim C.W. (2006). Deep femoral artery aneurysm presenting as lower limb swelling: a case report. Yonsei Med. J..

[7] Agha R.A., Borrelli M.R., Farwana R., Koshy K., Fowler A., Orgill D.P., For the SCARE Group (2018). The SCARE 2018 statement: updating consensus surgical CAse REport (SCARE) guidelines. Int. J. Surg..

[8] Perry M.O. (1993). Complications of missed arterial injuries. J. Vasc. Surg..

[9] Hartung O., Garcia S., Alimi Y.S., Juhan C. (2004). Extensive arterial aneurysm developing after surgical closure of long-standing post-traumatic popliteal arteriovenous fistula. J. Vasc. Surg..

[10] Ilijevski N., Radak D., Radevic B., Sagic D., Kronja G., Misovic S., Simic A. (2002). Popliteal traumatic arteriovenous fistulas. J. Trauma.

[11] Wang K.T., Hou J.Y., Hsieh J.J., Chou Y.S., Tsai C.H. (1998). Late development of renal arteriovenous fistula following gunshot trauma. Angiology.

[12] Banno H., Yamanouchi D., Fujita H., Nagata J., Kobayashi M., Matsushita M., Nishikimi N. (2004). External iliac venous aneurysm in a pregnant woman: a case report. J. Vasc. Surg..

